# An Innovative Transitions Model of Care for Delirium: “DDEFY Delirium” A Pilot Feasibility Randomized Trial

**DOI:** 10.1093/geroni/igab046.2596

**Published:** 2021-12-17

**Authors:** Ariba Khan, Marianne Klumph, Alexander Schwank, Sandy Hubatch, Jonny Macias Tejada, Colleen Galambos, Michelle Simpson, Michael Malone

**Affiliations:** 1 Advocate Aurora Health Care, Milwaukee, Wisconsin, United States; 2 Advocate Aurora Health, Milwaukee, Wisconsin, United States; 3 University of Wisconsin- Milwaukee, Milwaukee, Wisconsin, United States

## Abstract

In current standard practice, without a structured process for delirium follow up, older individuals and their family caregivers seemed to be lost, as they transitioned from hospital to home. The aim of this study was to pilot test a theoretical post-hospital model of care (DDEFY delirium) to mitigate the complications in patients who had hospital delirium. This is a pilot feasibility randomized controlled trial for patients with hospital delirium. The intervention was carried out by a delirium transitions nurse with personalized interdisciplinary team recommendations. DDEFY delirium intervention encompasses: Diagnose cognitive disorder; review Drugs; Educate patient/family; assess Function; Your health goals. During COVID-19 pandemic a virtual intervention group was created. Thus, three groups were analyzed: control, intervention, and virtual intervention. Among the 35 participants (mean age 80 years (SD10), 40% Black, 46% female), 40% had a diagnosis of dementia, mean Charles Deyo score was 6.4, mean number of medications 11.4 (3.2), and a mean anticholinergic medication burden was 2.4. The intervention group and virtual intervention group rates were: recruitment: 44.6 %vs8.8%, feasibility: 97%vs97%, fidelity:100%vs100%, 30-day readmission 28.6%vs0%, and 30-day ED visits: 0 vs.1. There were no differences in 30-day readmission rates between control vs intervention (p=1.0), control vs virtual intervention (p=.53), nor comparing all 3 groups (p=.49). The results of this pilot study determined that delivering DDEFY intervention to patients with delirium is feasible. Lessons learned from conducting this study will help us design a larger trial with modifications for older patients with delirium who transition from hospital to home.

